# Supplementary material for: Quantifying free behaviour in an open field using k-motif approach
Source: Sci Rep. 2019 Dec 27;9:19873. doi: 10.1038/s41598-019-56482-z (PMC6934846; doi:10.1038/s41598-019-56482-z)
Supplement: Supplementary file 1 — Supplementary Materials [file 41598_2019_56482_MOESM1_ESM.pdf]

Quantifying free behaviour in an open field using k-motif approach: Supplementary Materials

Marein Könings[a], Mark Blokpoel[a,b], Katarzyna Kapusta[c], Tom Claassen[a], Jan K. Buitelaar[c], Jeffrey C. Glennon[c], Natalia Z. Bielczyk[c,*]

[a]*Radboud University Nijmegen, Comeniuslaan 4, 6525 HP Nijmegen, the Netherlands*
[b]*Donders Centre for Cognition, Montessorilaan 3, 6525 HR Nijmegen, the Netherlands*
[c]*Department of Cognitive Neuroscience, Radboud University Nijmegen Medical Centre, Geert Groteplein Zuid 10, 6525 GA Nijmegen, The Netherlands*

Supplementary Material 1: Experimental datasets

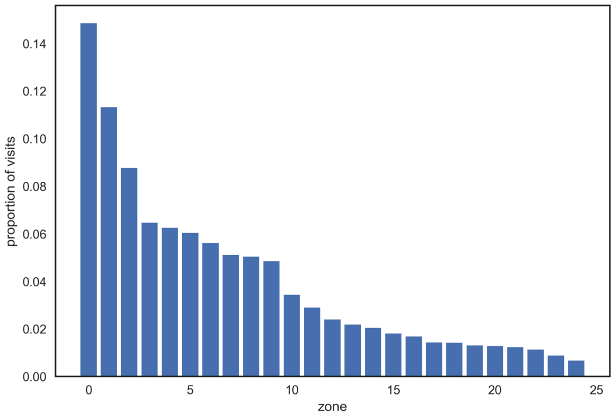

Supplementary Figure 1: Proportion of visits to each zone in the open field, in our datasets. The distribution is significantly non-uniform (Kolmogorov-Smirnov test at $p < 0.05$), which breaks the assumptions of the T-pattern method.

Supplementary Table 1: Descriptions of experimental variables recorded with Ethovision software

Variable	Unit	Description
Trial time	seconds	time since the animal was introduced into the open field environment
Recording time	seconds	time since the video recording was started
X centre	centimetres	x-coordinate of the animal's centre
Y centre	centimetres	y-coordinate of the animal's centre
X nose	centimetres	x-coordinate of the animal's nose
Y nose	centimetres	y-coordinate of the animal's nose
X tail	centimetres	x-coordinate of the animal's tail
Y tail	centimetres	y-coordinate of the animal's tail
Area	square centimetres	the amount of area covered by the body of the animal
Area change	square centimetres	the amount of body area that does not overlap with the body area of the previous measurement [1]
Elongation		a measure of how much the animal stretches its body out, between 0 and 1
Direction	degrees	the direction the animal is facing

Supplementary Material 2: Data preparation

The datasets were incomplete, which is often the case in translational psychiatry experiments. It was found that the experimental data contained missing values, non-uniform structure, redundancy and anomalous data. For each of these issues, a solution was proposed and implemented. Most solutions involve disregarding parts of the original data, but enough data remains to support the aim of the project, especially after augmentation (Section *Supplementary Material 3: Data augmentation*).

Missing values

Some sessions were missing while some other sessions were cut short, as a result of technical problems that occurred during the experiment. Lastly, at certain time points within sessions, some data was missing, leading to long contiguous periods missing data within a session. Specifically, there were some gaps of a spatial nature, occurring in multiple sessions, always at the same area in space (Supplementary Figure 2). In sessions affected by these gaps, whenever a subject passes through the affected area, a temporal gap in the data occurs. Since the spatial form of these gaps is roughly elliptical, these kinds of gaps are most likely caused by shadows or highlights on the surface of the open field. These features are caused by the computer

vision software determining the subject's position. The effect is less prominent in the *X centre* and *Y centre* variables, with most missing values occurring in the other *X* and *Y* variables.

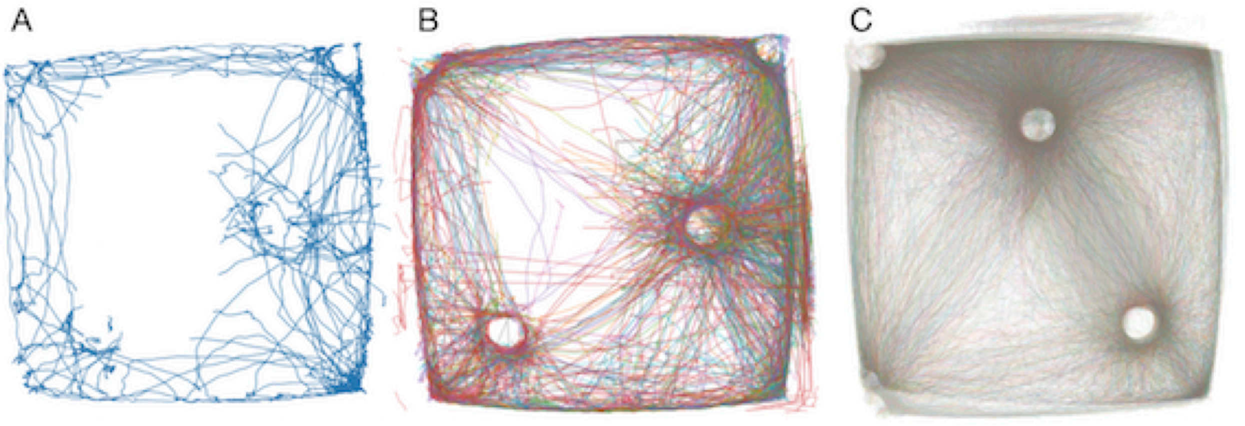

Supplementary Figure 2:**A**: An exemplary case of missing values of a spatial nature, a single session (this data was not used in the final study). A large area to the centre left of the open field is devoid of paths; all encroaching paths are cut off at the boundary of the area. **B**: The issue of missing values of a spatial nature, an overlay of a few sessions, with each subject marked with a different colour (this data was not used in the final study). The gap, although less pronounced, is also visible here, showing that it is a structural problem. **C**: An overlay of all movement data for all sessions from two different datasets: the dataset used in our study, and another dataset produced using the same experimental setup. Although the locations of objects and boundaries are aligned between sessions within datasets, there is a clear difference in these locations between datasets, as can be seen by the displacement effect in the image. The projections from the two datasets have been translated and rotated to achieve a best fit, but even so the difference is clear.

Multiple solutions to these problems were considered, most prominently either interpolating the data to fill the gaps, or complete removal of sessions containing missing data. Interpolation was difficult in this case, as this would require prior characterisation of the existing data, while characterisation was the point of the method. Hence, we decided to remove all the sessions containing missing data (Supplementary Table 2).

Non-uniformity

In this work, we had a selection of datasets to work on, coming from a few distinct experiments on the quinpirole model of obsessive-compulsive disorder in rats. However, the data displayed problematic non-uniformity between and within datasets, causing problems when comparing sessions. For example, the scale and orientation of the coordinate space might differ between datasets. These problems can largely be attributed to differences in camera position and angle at the time of recording. Because of perspective and lens distortion effects, these camera parameters have a large effect on the shape of the open field as projected into two-dimensional space. Although within any single session the camera is stationary, these effects cause problems when attempting to compare between sessions and datasets where the camera parameters differ (Supplementary Figure 2 C). For this reason, only a single dataset is used for the project, namely the dataset which contains the largest number of sessions, and also happens to display uniform camera parameters between sessions, was used for the final study.

An additional problem is caused by the fisheye effect (Supplementary Figure 2). An almost unpreventable effect of using a video camera, it causes the recorded footage to become warped. Since Ethovision 3.0 does not take this issue into account, the resulting data is also warped. Although the severity of the effect on

Supplementary Table 2: Sessions retained after pruning the dataset. The group to which each rat belongs ($s$aline or $q$uinpirole) is presented in the second row. The leftmost column presents the session number. The inner cells show the internal identifier for each rat-session combination.

rat	109	110	111	112	113	114	115	117
grp.	s	s	s	q	q	q	q	q
s1	4	5	6	7	8	9	10	12
s2	16	17	18	19	20	21	22	24
s3	28	29	30	31	32	33	34	36
s4	40	41	42	43	44	45	46	48
s5	52	53	54	55	56	57	58	60
s6	64	65	66	67	68	69	70	72
s7	76	77	78	79	80	81	82	84
s8	88	89	90	91	92	93	94	96
s9	100	101	102	103	104	105	106	108
s10	112	113	114	115	116	117	118	120

further data analysis is not obvious, it would be good to try and revert the warping effect in the resulting data. However, that has not been attempted in this project.

Anomalies

In some sessions, some anomalous sudden jumps seemingly made by the subject over large distances from one location to another (the 'teleportation effect') can be found. Sometimes these anomalies even seem to transport the subject to locations outside the regular bounds of the open field (e.g., Supplementary Figure 2 A, B). These jumps almost always come in pairs, where the subject is transported to an anomalous location and then transported back to the original area after a fraction of a second. It seems that these anomalies can be attributed to errors made by the computer vision software when extracting the movement data from the raw video footage. Anomalies may be detected by looking for position change between two adjacent time points that would be impossible for an actual rat to accomplish - assuming a top speed of 9.6 km/h for rats [2], 93 such event occur in the dataset (0.002% of all time points).

One elegant solution to the problem of anomalies would be to detect these anomalies, remove the data between two instances of 'teleportation' so that the period of anomalous movement is no longer present, and then reconstruct the missing values by means of interpolation. However, as discussed in Section *Missing values*, interpolation is difficult and can cause that the classification problem becomes ill-posed. Therefore, since only such a small percentage of all data is affected with this problem, we decided to neglect it in this work.

Redundancy

Since the original data contains a large number of variables, some of which describe phenomena that seem closely intertwined, there is redundancy in the data. For example, the location of the subject's head can be predicted from the location of the subject's torso with a high accuracy. It might then be possible to remove certain variables from the data, without losing predictive power in the classification. Therefore, we calculated pairwise correlations between all of the variables in the complete dataset (preprocessed with the previous step) with use or Pearson's r (Supplementary Figure 3 A).

Going further, the set of variables can be pruned so that only those variables remain which do not significantly correlate with any earlier variable. Given two variables $v, w \in V$, the variable w is 'earlier' than v ($w < v$) if w precedes v in the standard order of variables. The resulting set of variables V' is defined in Equation 1.

$$V' = \{v \in V \mid \neg\exists_{w \in V} w < v \text{ and } R(v, w)\} \quad (1)$$

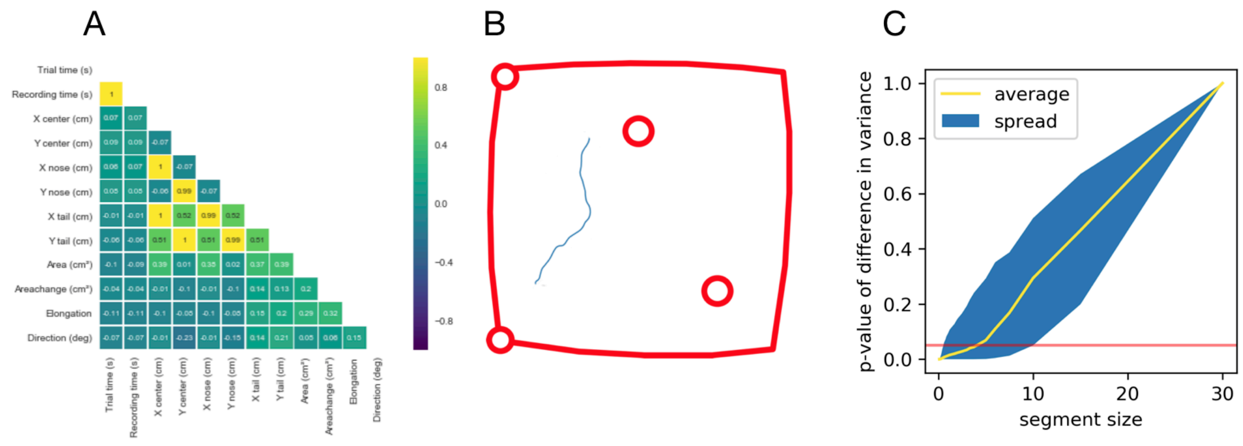

Supplementary Figure 3: **A**: Correlations between variables recorded by Ethovision. **B**: a very small segment of an open field session has too little features to use for describing behaviour. **C**: Data segmentation. As the segment size decreases, so does the p-value of the the difference in variance as compared to the original segment size (30 minutes). The red line indicates a threshold $p = 0.05$. The yellow line presents the average p-value over all variables in V. The blue area shows the spread of the p-value for all variables.

where $R(v, w)$ - Pearson's r coefficient between variables w and v. This pruning technique ensures that out of all sets of inter-correlating variables, only one remains, namely the first in the order as defined.

Supplementary Material 3: Data augmentation

Given the ultimate goal of creating a quantification method and evaluating it using classification, it is important to have sufficient sessions for effectively training a classifier. Therefore, since the data undergoes significant reduction in the preprocessing stage, the possibility of data augmentation was considered. In machine learning, data augmentation is the process of extending the available data to be more multitudinal or vibrant, before using it as input for learning algorithms [3].

In this work, we augment the data. Given that each session is reduced to a single sessions (set of features), the amount of sessions is equal to the amount of sessions. However, each session has a duration of half an hour but the features are observables which be measured within far less time. Thus, each session is split into a number of segments, which can then all be used for feature extraction, yielding a larger number of sessions.

Optimal split of the data into segments is not an trivial problem: if the sessions are split into too many segments, each segment will be so small for the proper feature estimation, while at the same time, the more splits, the more sessions. Thus, there is a trade-off between the length of the data in each segment, and the number of segments (Supplementary Figure 3 B, C).

Two methods for determining the optimal segment size are evaluated. The first method is based on the variance of the data in a segment as the segment size changes: as the segment size decreases, the variance in the data must decrease as well. The significance of this decrease from the original is calculated. Then, based on significance, the optimal segment size can be determined as a segment as small as possible while not displaying a significant difference in variance from the original data.

The second method for determining the optimal segment size is based on prior literature [4, 5, 6]. Many rodent experiments involve measuring the frequency of certain behaviours displayed by the animal, such as grooming or marble-burying. If the frequency of such behaviours can be determined from the literature, especially with regards to OCD behaviour, then this would be a good indication of how small segments can be while retaining the possibility to detect these behaviours in each segment.

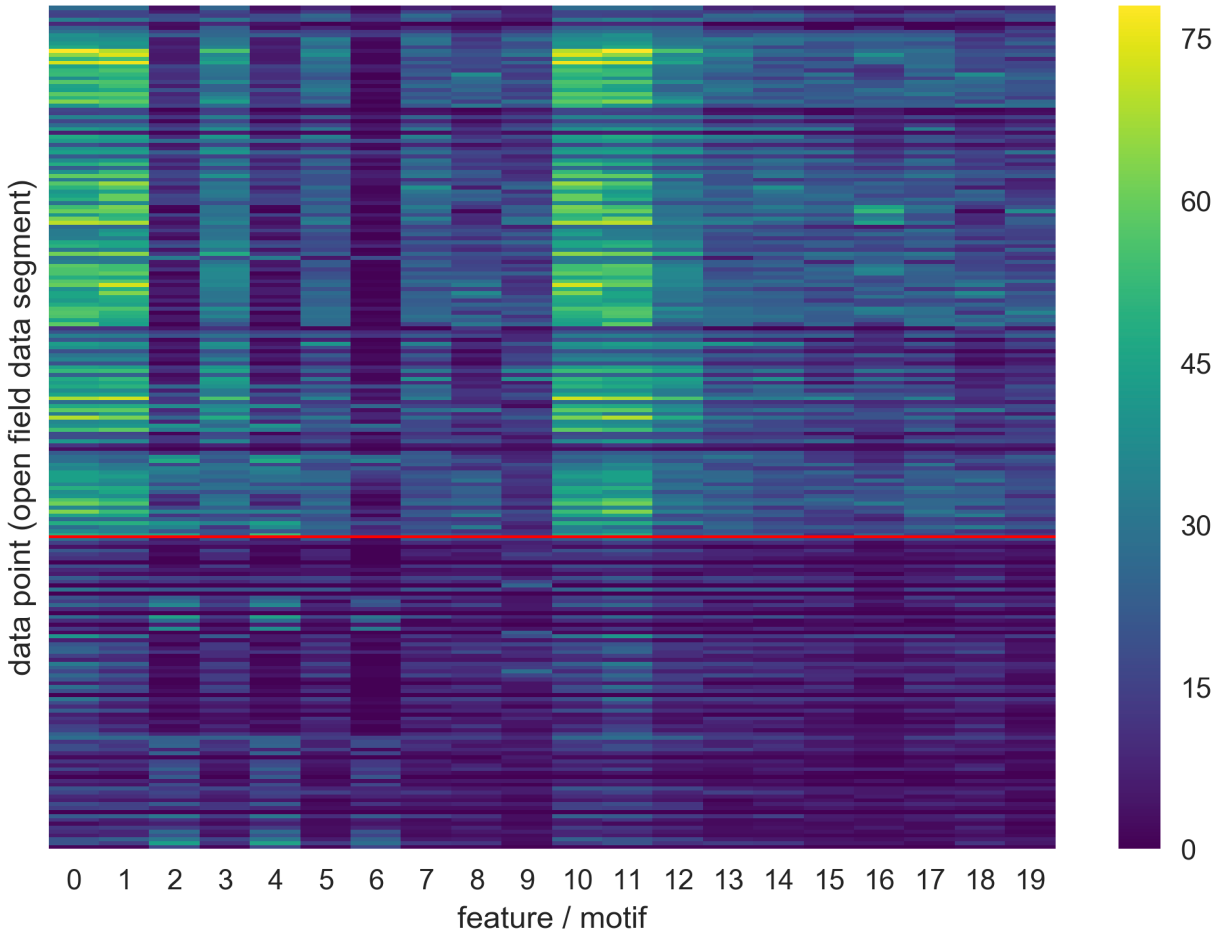

Supplementary Figure 4: A summary of all feature vectors for all sessions, using the I_2 measure. On vertical axis, all the data segments are listed (multiple sessions per subject). The units of the colour key are arbitrary; colours symbolise total count of the given motif in the given session (this is why the colour scale is between 0 and 75, which was the maximal count). Note that sessions are separated by class, as indicated by the red line, with the quinpirole group at the top and the control group at the bottom (there are more sessions in total in the quinpirole group than in the control group because of the data cleaning).

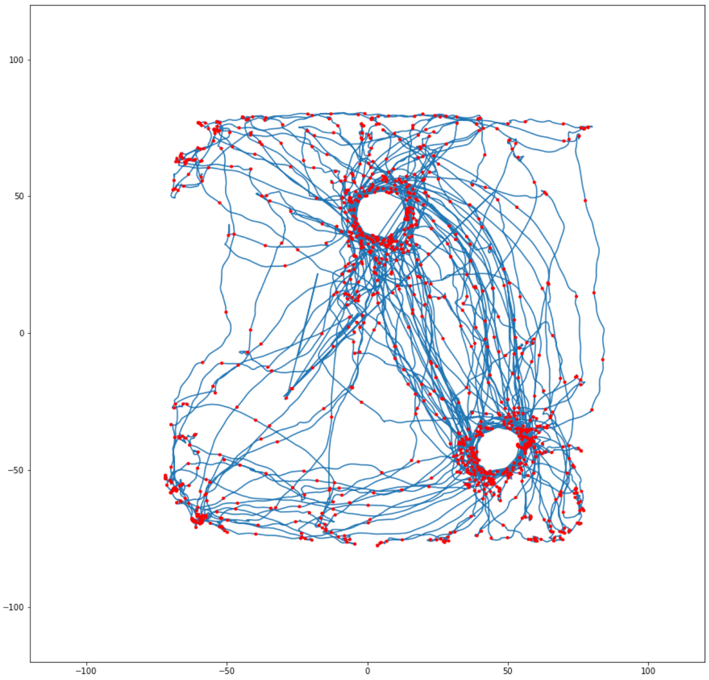

Supplementary Figure 5: An exemplary dynamics throughout the session for exemplary subject (subject 113, session 1). The red dots denote data points every 1.0 [s] (every 40 frames). As visible on the picture, the subject not only recurrently returns towards the objects but also spends the most time around them.

References

[1] Juszczak, G. R., Lisowski, P., Śliwa, A. T. & Swiergiel, A. H. Computer assisted video analysis of swimming performance in a forced swim test: simultaneous assessment of duration of immobility and swimming style in mice selected for high and low swim-stress induced analgesia. *Physiology & Behavior* **95**, 400–407 (2008).

[2] Wolfram Alpha LLC. Wolfram|Alpha (2009).

[3] Fawzi, A., Samulowitz, H., Turaga, D. & Frossard, P. Adaptive data augmentation for image classification. In *Proceedings of the Image Processing (ICIP), 2016 IEEE International Conference*, 3688–3692 (Ieee, 2016).

[4] Szechtman, H. *et al.* Compulsive checking behavior of quinpirole-sensitized rats as an animal model of Obsessive-Compulsive Disorder (OCD): form and control. *BMC Neuroscience* **2**, 4 (2001).

[5] Sesia, T., Bizup, B. & Grace, A. A. Evaluation of animal models of obsessive-compulsive disorder: correlation with phasic dopamine neuron activity. *Int J Neuropsychopharmacol* **16**, 1295–307 (2013).

[6] Shmelkov, S. V. *et al.* Slitrk5 deficiency impairs corticostriatal circuitry and leads to obsessive-compulsive-like behaviors in mice. *Nature medicine* **16**, 598–602 (2010).
